# Supplementary figures and images for: Designing host-associated microbiomes using the consumer/resource model
Source: mSystems. 2024 Dec 9;10(1):e01068-24. doi: 10.1128/msystems.01068-24 (PMC11748559; doi:10.1128/msystems.01068-24)

(A)

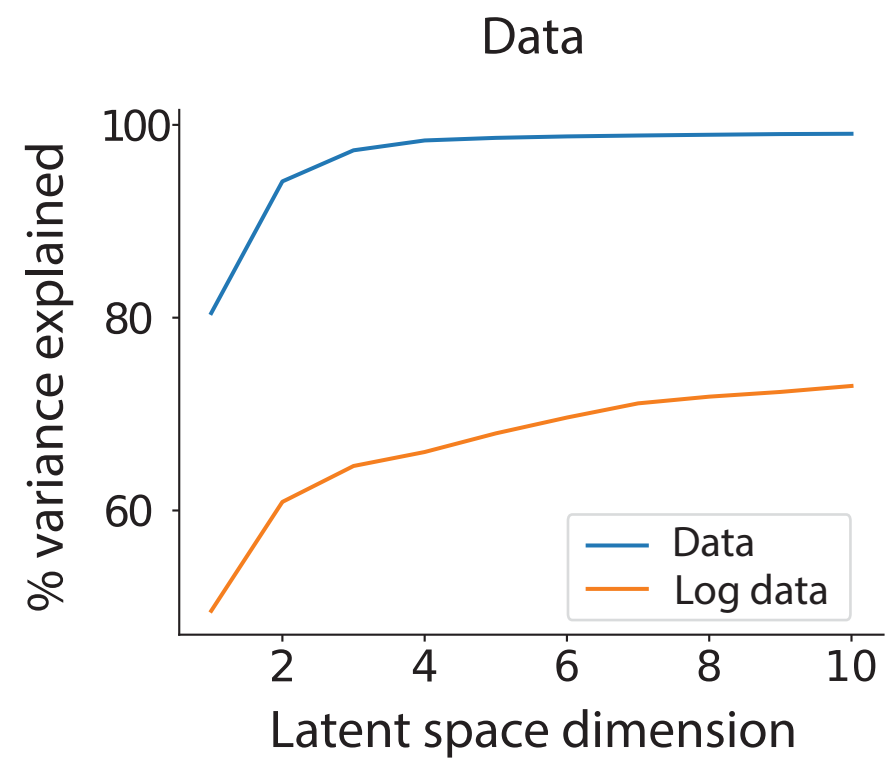

(B)

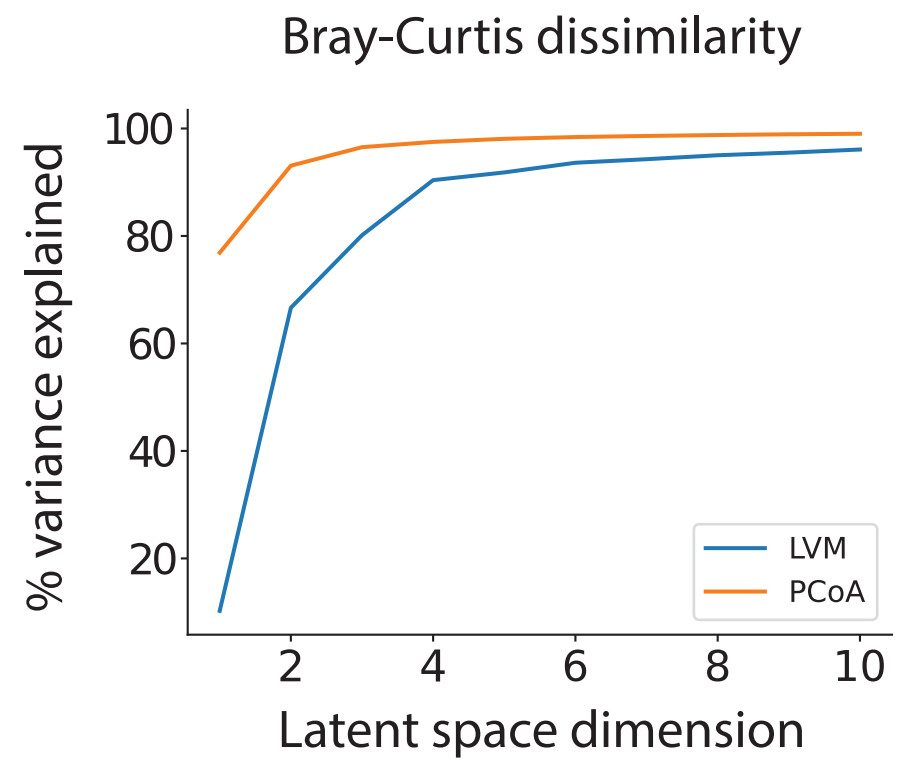

(A)

Jensen-Shannon divergence

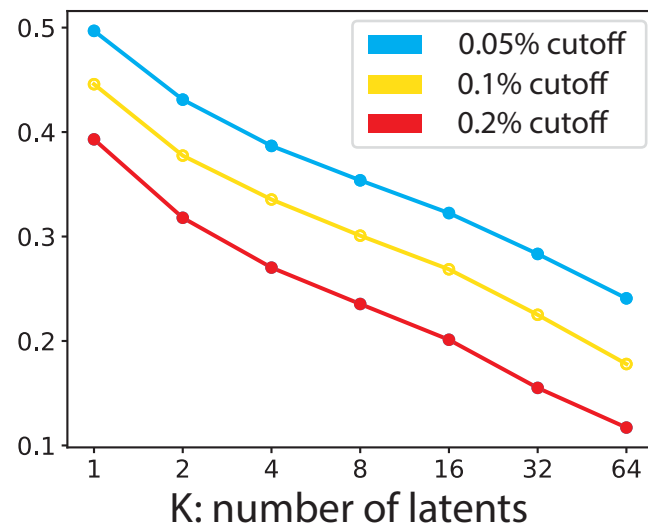

(B)

Bray-Curtis dissimilarity

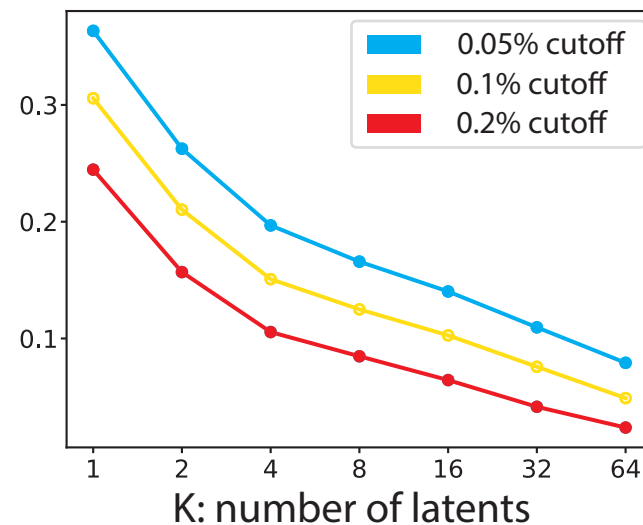

(C)

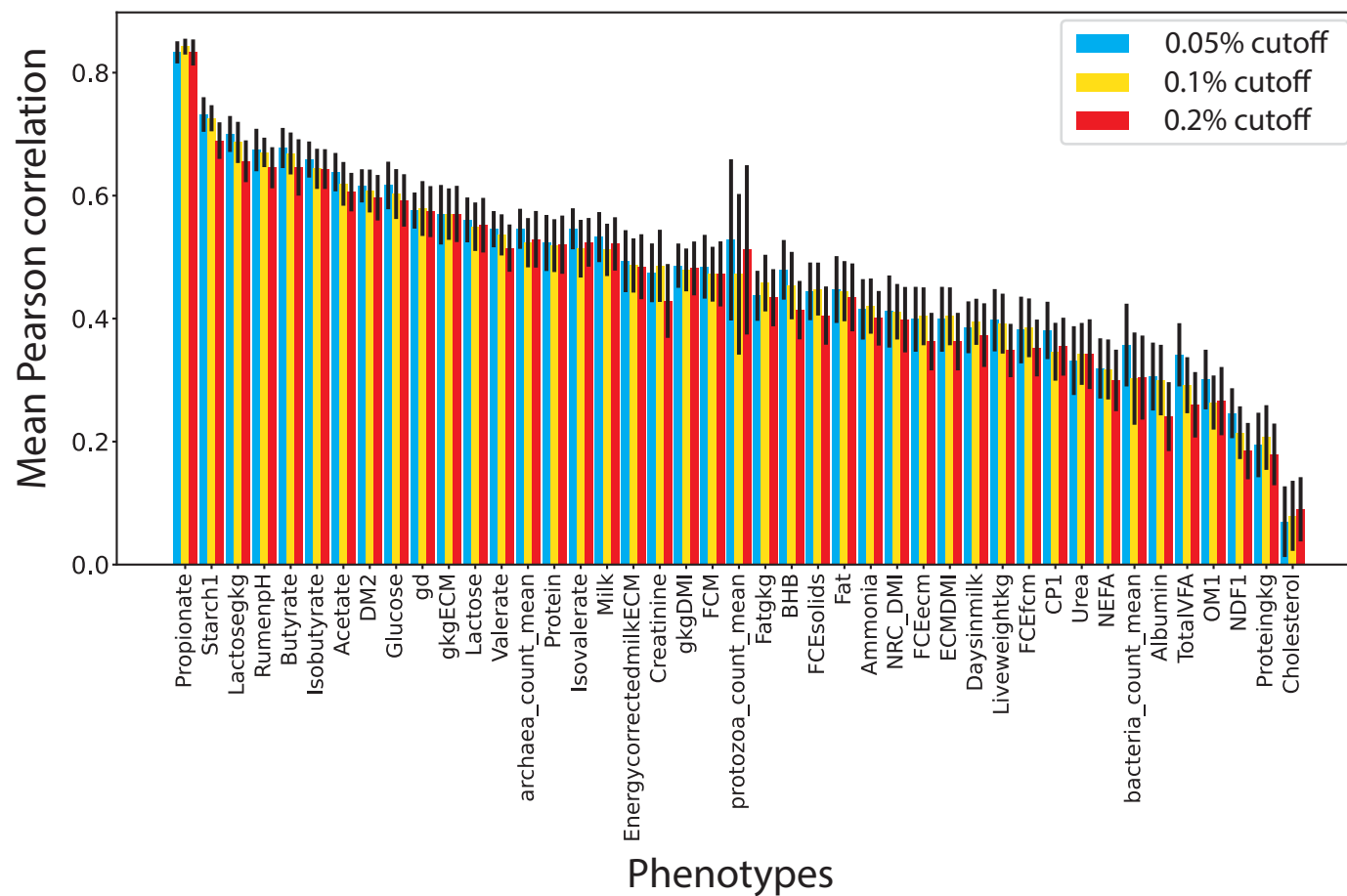

(A)

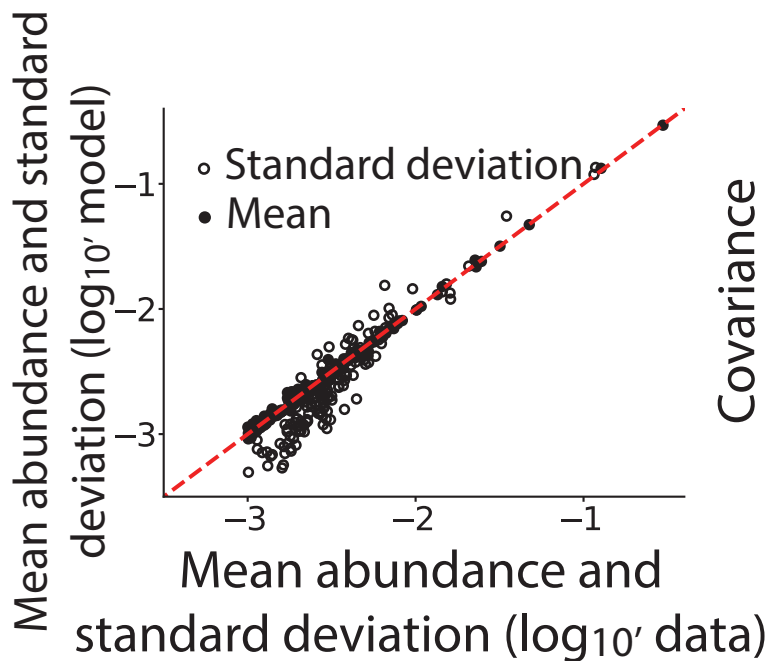

(B)

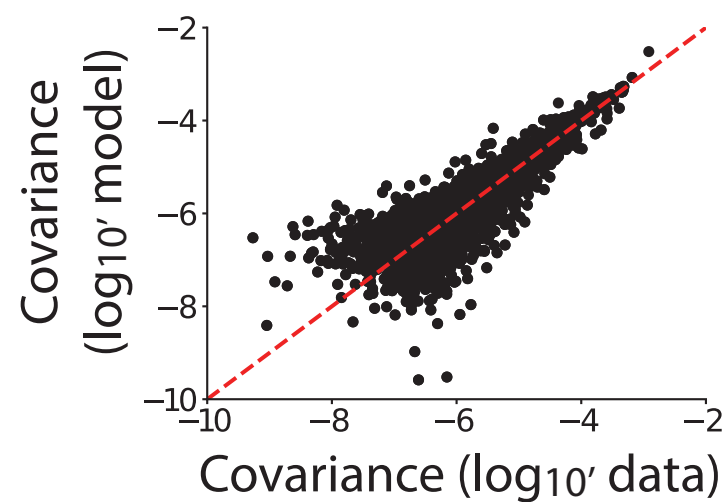

(C)

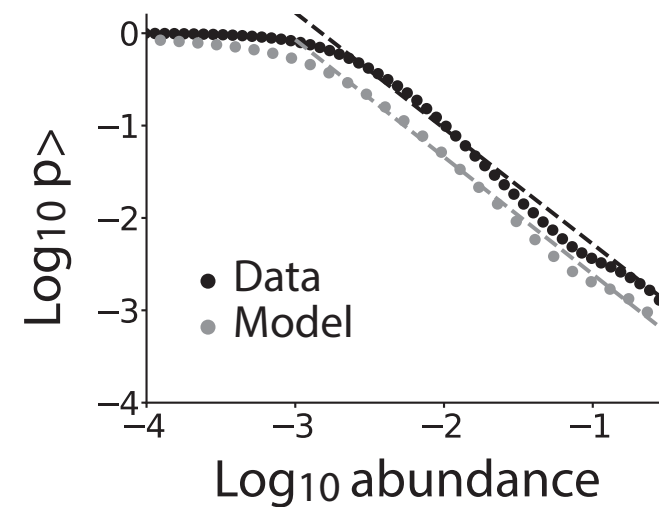

(D)

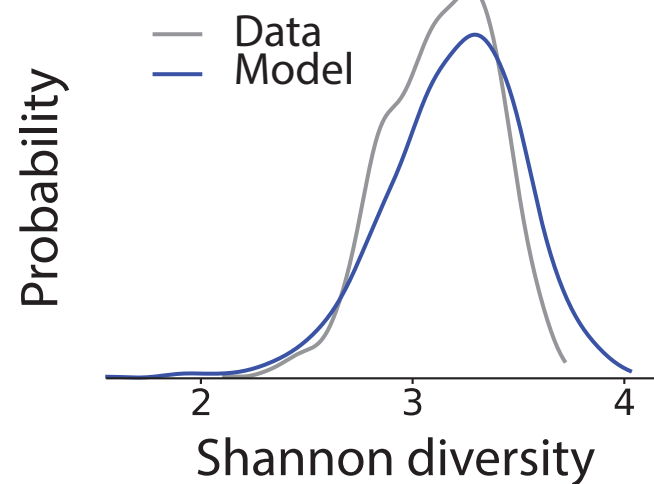

(E)

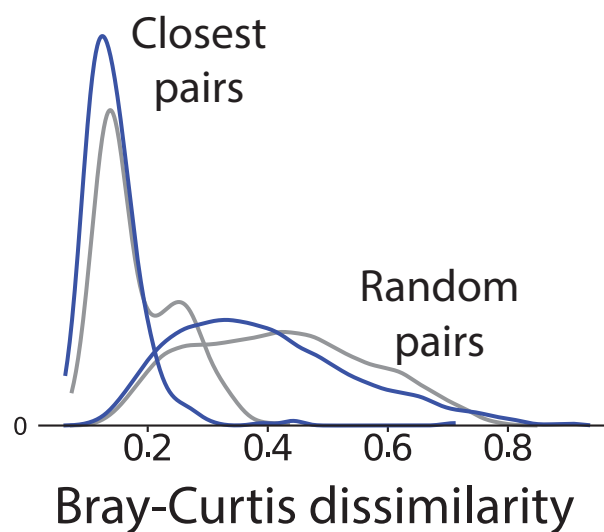

(F)

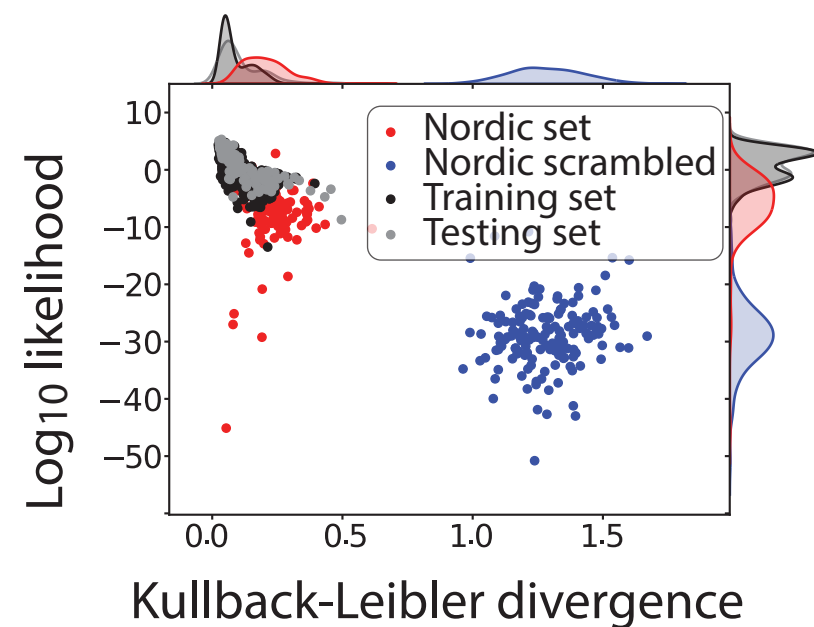

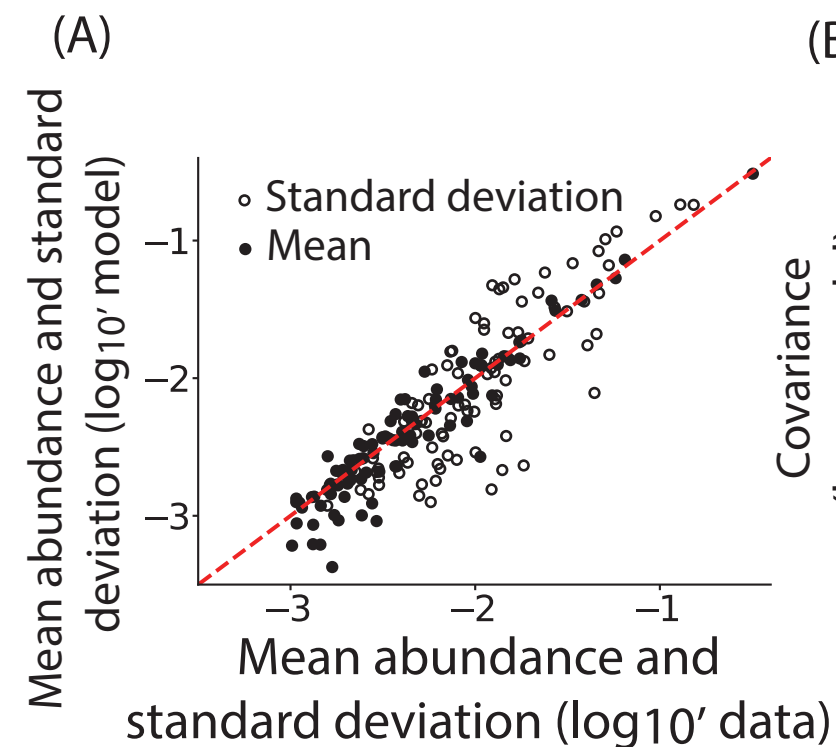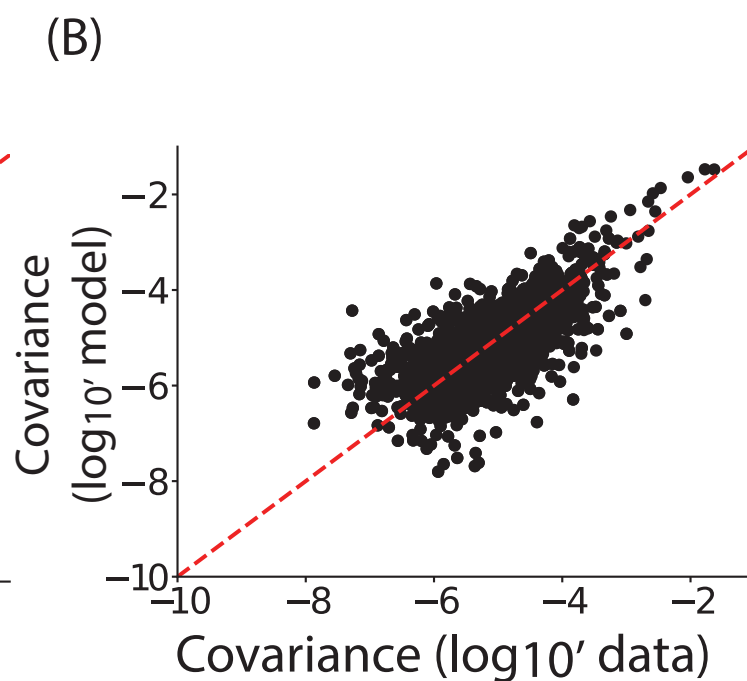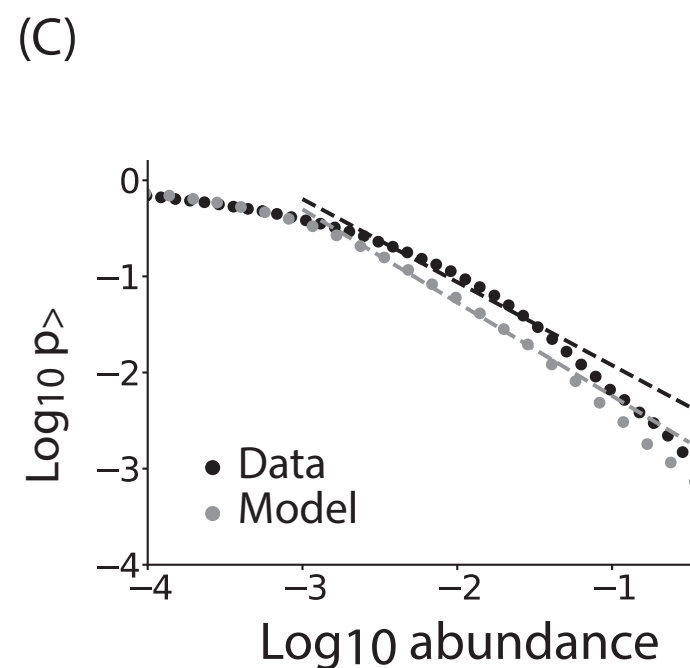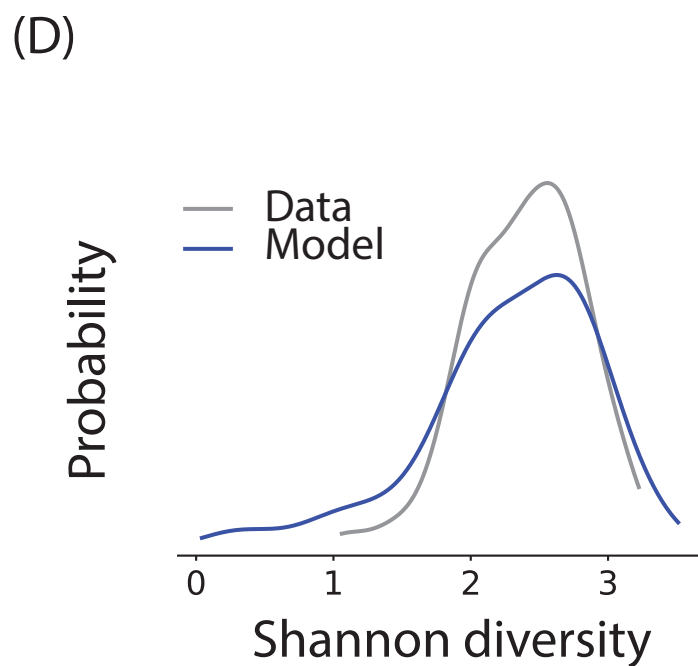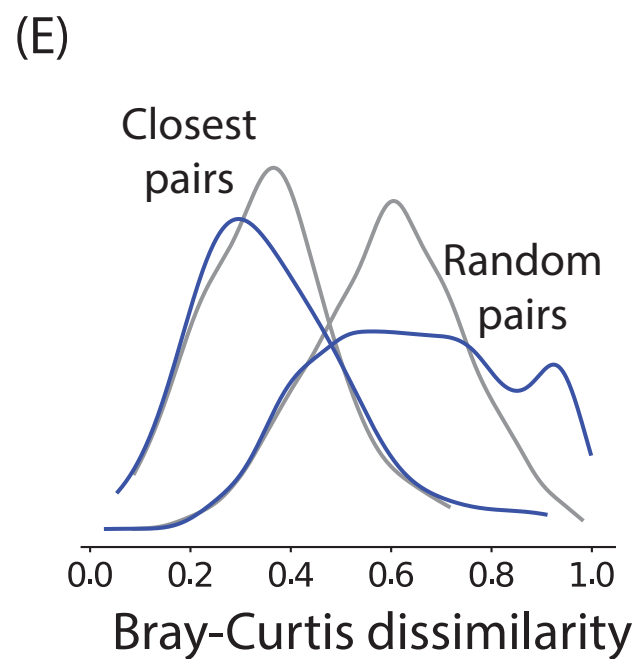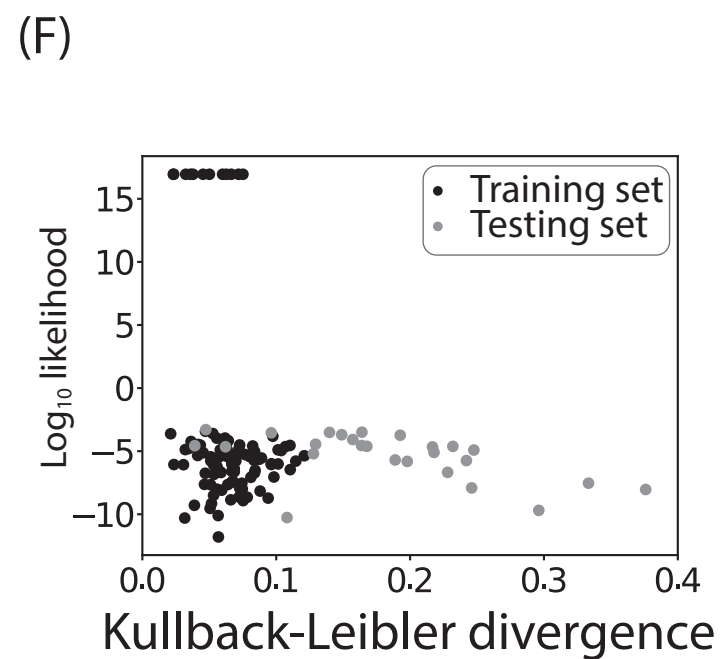

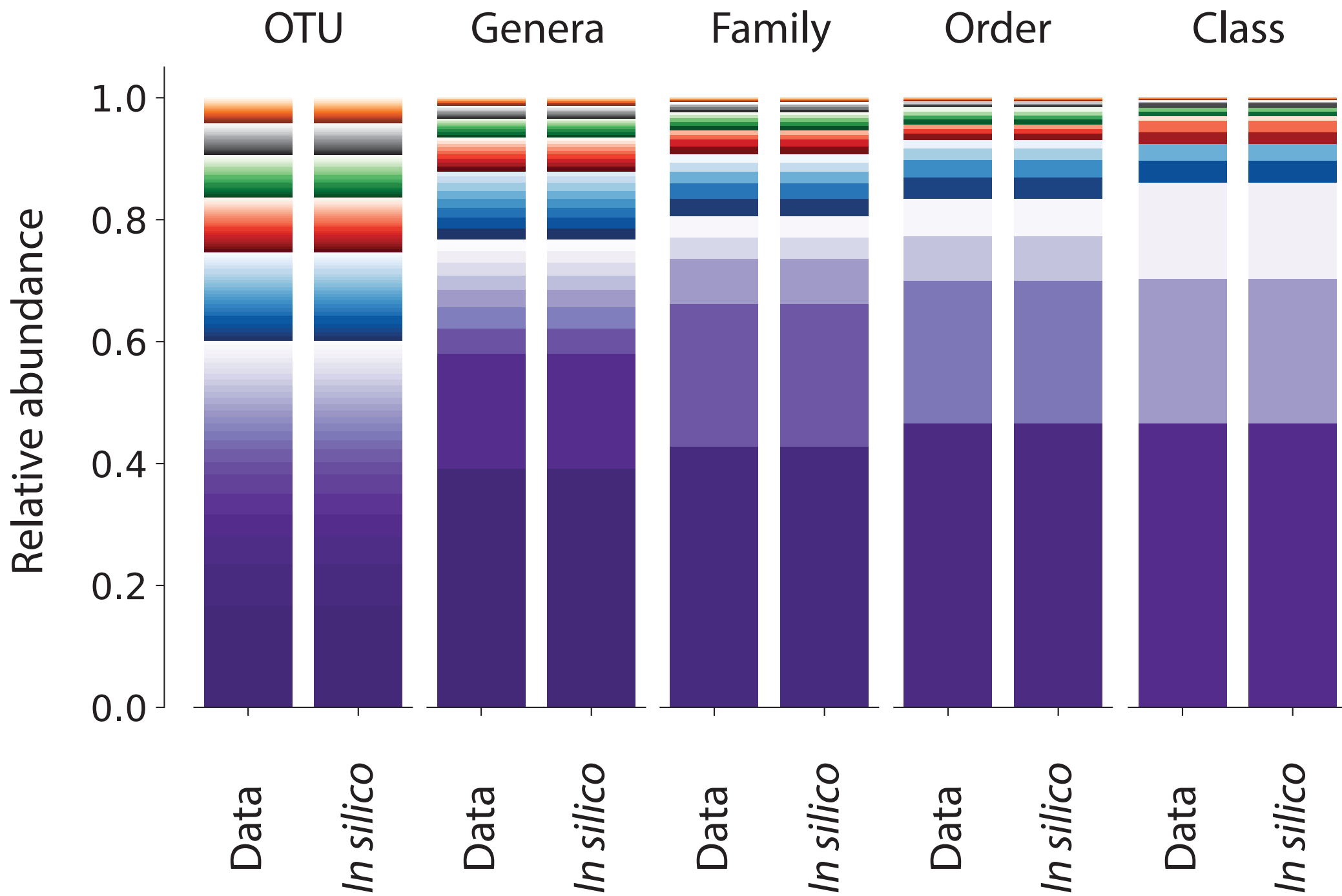

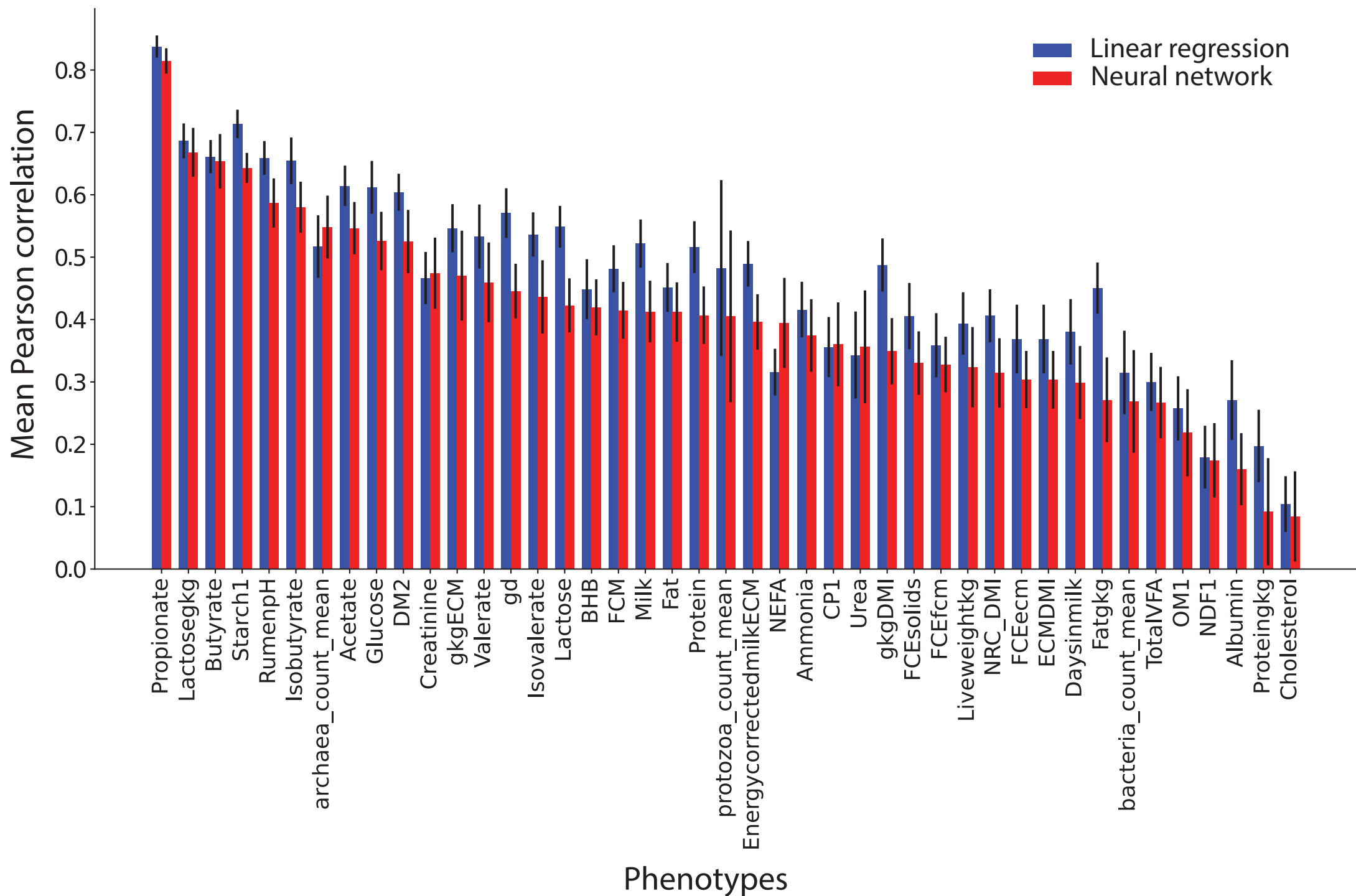

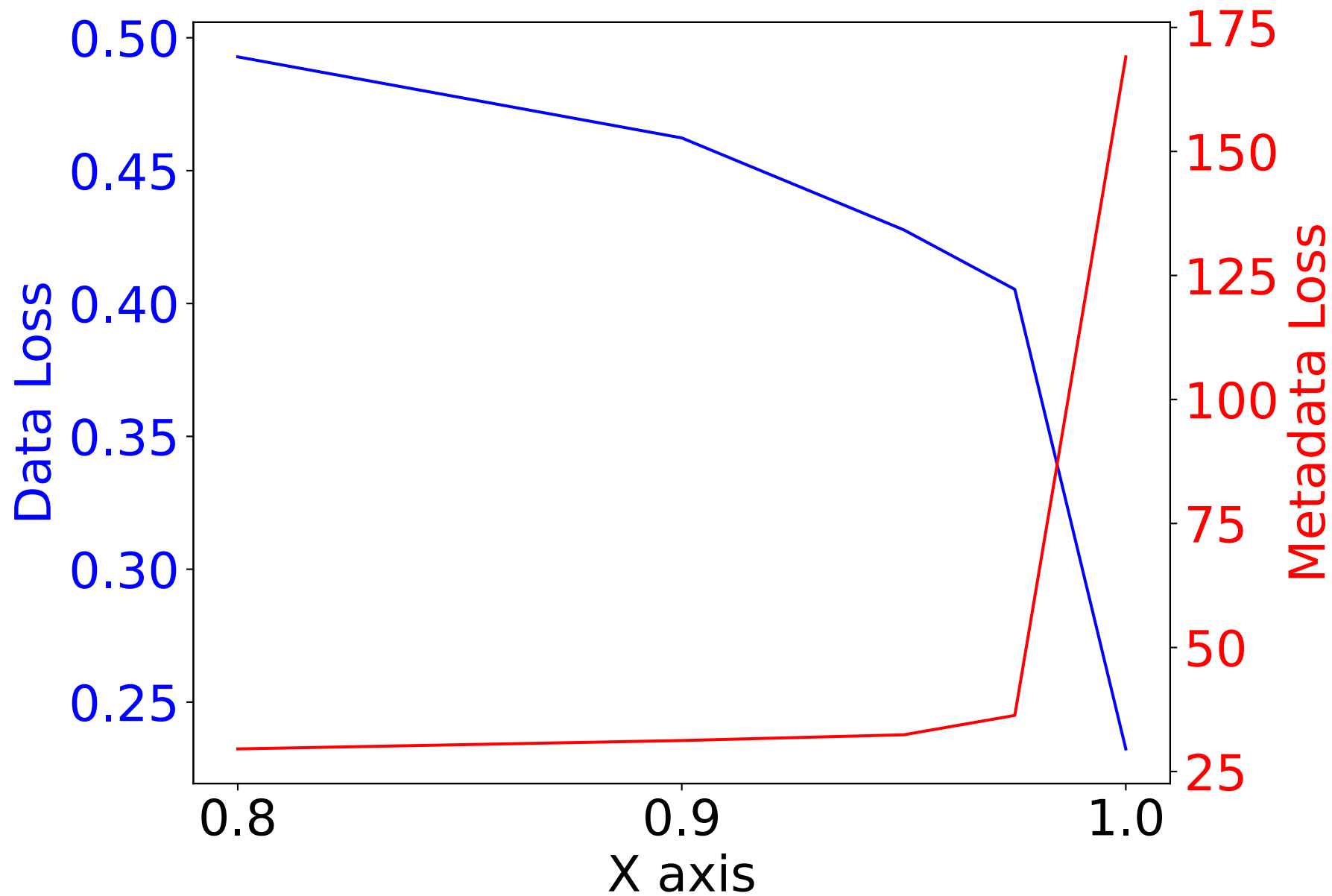

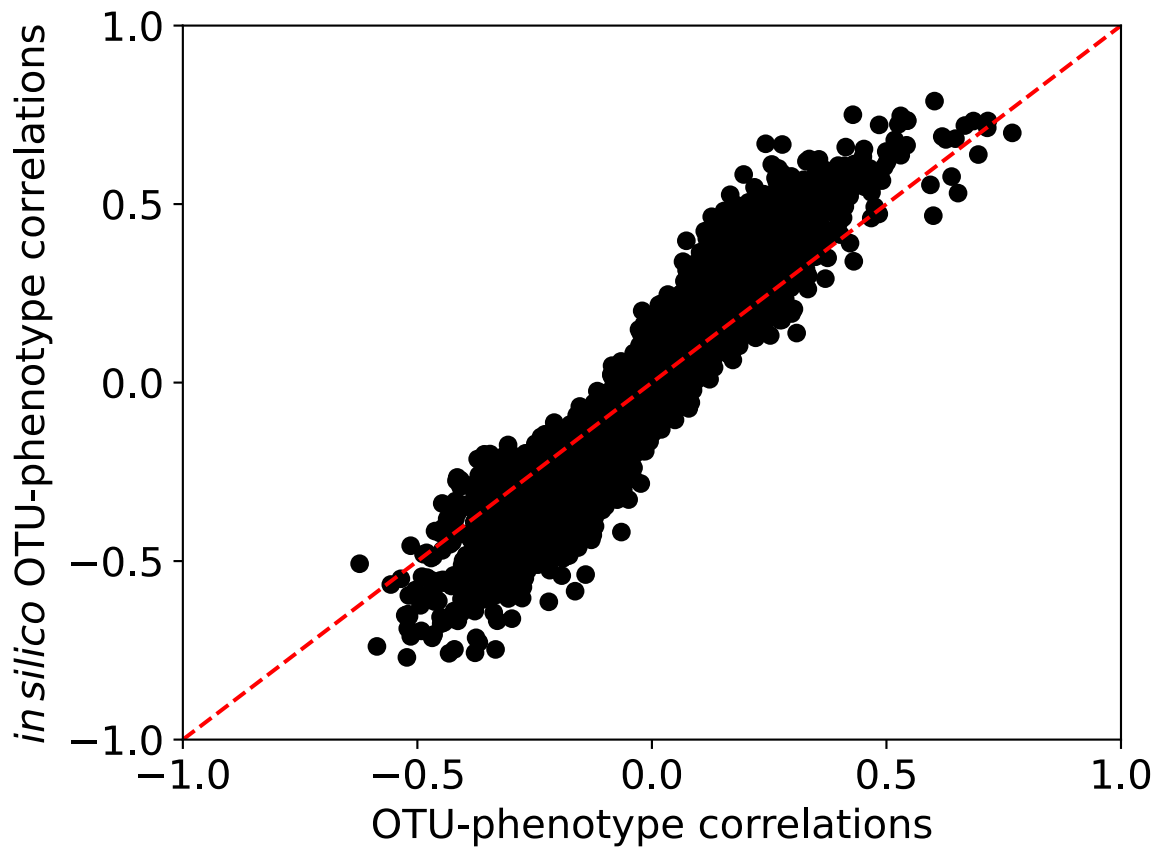

(A)

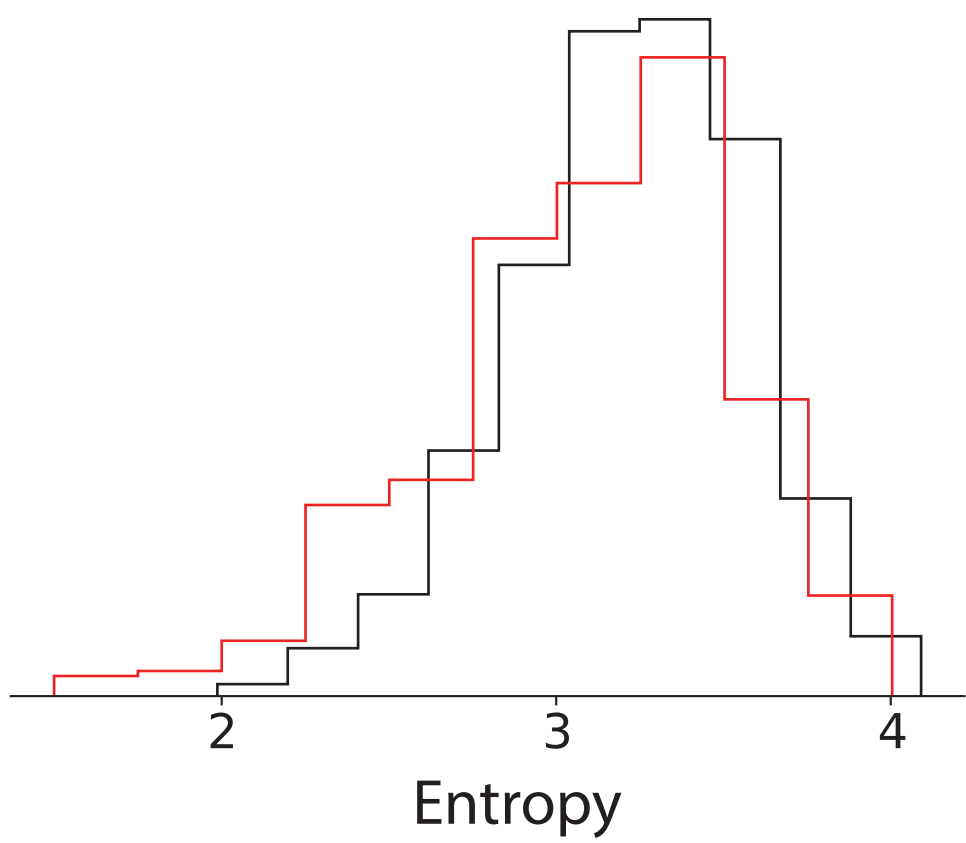

(B)

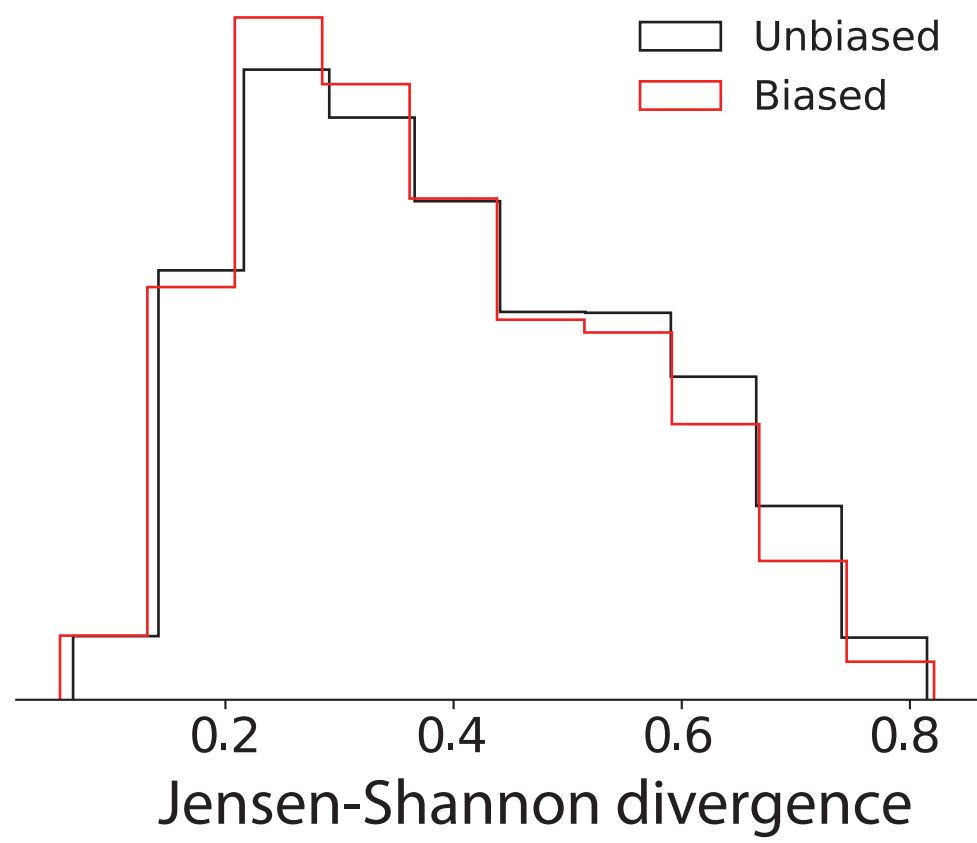

Supplement: Supplemental Figures — Figures S1 to S9. [file msystems.01068-24-s0001.pdf]
